# Ultra-Processed Food Consumption Is Associated With Poor Diet Quality and Nutrient Intake Among Adolescents in Urban Slums, Kenya

**DOI:** 10.3389/ijph.2024.1607891

**Published:** 2025-03-05

**Authors:** Milkah N. Wanjohi, Gershim Asiki, Calistus Wilunda, Michelle Holdsworth, Rebecca Pradeilles, Linda Simon Paulo, Nelson Langat, Dickson A. Amugsi, Simon Kimenju, Elizabeth W. Kimani-Murage, Kerstin Klipstein-Grobusch

**Affiliations:** ^1^ Julius Global Health, Department of Global Public Health and Bioethics, Julius Center for Health Sciences and Primary Care, University Medical Center Utrecht, Utrecht University, Utrecht, Netherlands; ^2^ African Population and Health Research Center (APHRC), Nairobi, Kenya; ^3^ UMR MoISA (Montpellier Interdisciplinary Centre on Sustainable Agri-Food Systems), University Montpellier, CIRAD, CIHEAM-IAMM, INRAE, Institut Agro, IRD, Montpellier, France; ^4^ Muhimbili University of Health and Allied Sciences, Dar es Salaam, Tanzania; ^5^ Kula Vyema Centre of Food Economics, Kiambu, Kenya; ^6^ Division of Epidemiology and Biostatistics, School of Public Health, Faculty of Health Sciences, University of the Witwatersrand, Johannesburg, South Africa

**Keywords:** ultra-processed, overweight, obesity, double burden of malnutrition, urban, slum, diet-quality, Kenya

## Abstract

**Objectives:**

To assess the caloric contribution of ultra-processed foods (UPFs), factors associated with UPFs energy intake and investigate the relationship between UPFs energy intake, diet quality and nutrient intake among adolescents in urban slums, Kenya.

**Methods:**

A cross-sectional household study amongst adolescents (10–19 years, N = 621) collected socio-demographic and dietary intake data. Global Diet Quality Score (GDQS); mean and percentage total energy intake (%TEI) from UPFs; and nutrient intakes were computed. Regression analysis assessed the factors associated with UPFs energy intake, and the association between %TEI from UPFs and diet quality.

**Results:**

Mean daily energy intake was 1,604 kcal (±550), 25.2% from UPFs. Higher leisure screen time (≥2 h/day) [OR = 1.9 (1.2–3.1)] was associated with UPFs energy intake. Household wealth index (quintile five vs. one) [OR = 2.6 (1.3–6.0)] was associated with non-UPFs energy intake. UPFs (%TEI) was inversely associated with GDQS score (quartile four vs. one) [β = −2.9 (−3.4 to −2.1)]. Adolescents with higher %TEI from UPFs (quartile four) had highest total energy, total fat and saturated fat; and lowest protein, fibre, iron, calcium and zinc intake.

**Conclusion:**

UPFs contribute substantially to adolescents’ energy intake and are linked to poor diet quality and nutrient intake.

## Introduction

The double burden of malnutrition (DBM) among adolescents, characterised by increasing overweight and obesity and a slow decline in undernutrition (stunting and wasting), is a major concern in low-income countries [1]. Unhealthy diets, including ultra-processed foods (UPFs) and foods that are high in fat, salt and sugar are a key driver of DBM [2, 3]. Rapid urbanisation is associated with changes in the food systems, characterised by increased availability of cheap and convenient unhealthy foods for the needs of the burgeoning urban population [4].

The NOVA classification system is the most widely used method for classifying the healthiness of food, based on the level of processing [5] and generally categorises foods into four groups: 1) unprocessed or minimally processed foods, 2) processed culinary ingredients, 3) processed foods and 4) UPFs [6]. UPFs comprise the least healthy food group in the NOVA classification, and their consumption is associated with the double burden of malnutrition (DBM) [3]; poor diet quality including high levels of salt, sugar and saturated fats [7–9], and low levels of protein, iron, vitamins and fibre [10–12] in adolescents. UPFs are also associated with excessive weight gain and increased risk of overweight, obesity, non-communicable diseases (NCDs) [13], metabolic [14] and cardio-metabolic syndromes [15]. Furthermore, a review of food systems in low and middle income countries including Africa, documents the existence of locally produced food/snacks resembling UPFs, although they are mainly not pre-packaged and often supplied through informal food vendors as ready to eat street fast foods, especially in urban areas [4, 16]. Similar to UPFs, such local snacks contain high levels of unhealthy saturated and trans fats, sugar, salt, highly refined carbohydrates [4, 17] and their frequent consumption is associated with poor diet quality and non-communicable diseases [18, 19].

Global and national healthy diet guidelines and key messages encourage consumption of minimally processed foods, and discourage consumption of foods that are high in fat, salt and sugar [20, 21] for optimal health and prevention of overweight/obesity and diet related diseases. The Kenyan guidelines for healthy diets further encourage consumption of foods rich in iron, zinc and calcium by adolescents [20]. Despite this, the burden of overweight and obesity is rising alongside that of chronic undernutrition and micronutrient deficiencies among adolescents in the sub-Saharan region [22, 23]. In Kenya, 43% of adolescent boys are underweight [24]. In addition, overweight/obesity prevalence among girls has increased from 8% in 2003 to 13% in 2022 [24]. In the Kenya National Micronutrient Survey, about 80% of adolescents (10–14 years) were zinc deficient while 27% of pregnant adolescents were anaemic or iron deficient [25].

Evidence on the dietary practices including UPFs consumption by adolescents in Kenya and sub-Saharan Africa is limited [26, 27], available literature mainly focus on food group (e.g., fruit and vegetable) consumption, and dietary diversity [28]. This study aims to address this evidence gap by 1) assessing the caloric contribution of UPFs to adolescents’ daily energy intake, 2) identifying factors associated with UPFs energy intake and 3) investigating the relationship between UPFs energy intake, diet quality and nutrient intake, among adolescents in urban slums in Kenya.

## Methods

### Study Design and Setting

A cross-sectional household survey of adolescents aged 10–19 years living in three major urban slums in Nairobi (*Mathare*, *Korogocho*, and *Viwandani*) was conducted from August to December 2021. *Mathare* slum is the second largest and one of the oldest slums in Kenya, with an estimated population density of 68,941 persons/km^2^ [29]. *Korogocho* slum is the fourth largest in Nairobi. It has an estimated population density of 100,000 persons/km^2^, with a longer mean slum residency (14 years) and a higher prevalence of chronic poverty and non-migrants (born in the slum) compared to Viwandani [30]. *Viwandani* slum is the smallest of the three with an estimated population density of about 12,825 persons/km^2^. It is located in the main industrial area, has a shorter mean slum residency (8 years) and has a relatively higher social economic status (SES), the lowest unemployment rate and lowest prevalence of chronic poverty compared to *Mathare* and *Korogocho* [31]. The three slums are generally characterised by poor housing and congestion; inadequate infrastructure including health, education, water and sanitation; high levels of violence, crime and insecurity; high unemployment and poverty rates and food insecurity [30, 32]. The African Population and Health Research Center operates the Nairobi Urban Health and Demographic Surveillance System (NUHDSS) in *Korogocho* and *Viwandani* slums, through which health and demographic data are collected routinely from about 79,000 individuals (aged 0–105 years) living in 25,000 households [30]. This study was nested within the NUHDSS and a larger *Healthy Food Africa* (HFA) project [33]. To obtain the sampling frame for this study, the NUHDSS census was used in Korogocho and Viwandani, while a separate household listing was conducted in Mathare.

### Sample Size Estimation and Sampling

Sample size was calculated using Cochrane’s formula for estimating sample size for proportions, using the documented prevalence of overweight/obesity among school going adolescents in Nairobi as 17.6% [34], and taking into account a level of precision of 5%, with 95% confidence level. Adjusting for a 20% non-response rate yielded a sample size of n = 327 (∼330) adolescents. Two age strata of younger (10–14 years) and older adolescents (15–19 years) were powered independently. A total sample size of 660 for both age groups was therefore estimated. A list of all households with eligible adolescents was obtained from the NUHDSS data for Korogocho and Viwandani and a household listing from Mathare. In the household listing, community health promoters visited all households in the slum, listing down all eligible adolescents in each household. Simple random sampling was then used to select a sample of 660 adolescents, proportionate to the number of eligible adolescents in each slum.

### Data Collection

#### Socio-Demographic Characteristics

A structured interviewer-administered questionnaire was used to collect adolescents’ socio-demographic information, including individual level [*age, sex, cultural background (ethnicity), self-reported leisure screen time* (e.*g., TV, and phone, computer, social media, and video games*)], household (*wealth index*) and neighbourhood (*slum of residence, duration in the slum*) characteristics. Household asset ownership was obtained from the adolescents’ primary caregiver, from which a household wealth index was computed using principal component analysis and categorised into household wealth index quintiles [35].

#### Anthropometric Measurements (Nutritional Status)

Anthropometric measurements (height and weight) were taken using standardised procedures, barefoot with minimum clothing [36]. Two measurements were taken for both height and weight and the average obtained. Height-for-age (HAZ), and BMI-for- age Z-scores (BMIAZ) were computed using the WHO anthros Stata macros (2007) [37]. The Z-scores were then classified as thin (BMIAZ < −2), overweight (BMIAZ >+1 and <+2), obese (BMIAZ > +2 BMI) and stunted (HAZ < −2), based on the WHO 2007 growth reference [37]. Overweight/obesity were combined as one group due to a small sample in the obesity group.

#### Dietary Data Collection

Dietary data were obtained through multiple 24-hour (24-h) open recall interviews, using the multiple pass method [38]. To capture intra-individual variability in dietary intake, repeat 24-h recalls were conducted on two non-consecutive days, representing one weekday and one weekend, within 2 weeks. Information on each of the food items consumed including the eating time and details of the food items (food type, ingredients of mixed dishes and brand names of commercially produced foods, and cooking method) and the amount consumed were collected. The food portion sizes and amounts consumed were estimated with the aid of the Kenya adolescent photographic food atlas [39], which contains photographic estimates of household measures, quantities and weights of foods that are commonly consumed by adolescents in urban settings in Kenya. The average (mean) amount and energy intake for both days was computed for subsequent analysis.

#### NOVA Food Classification

The NOVA food classification system [6] was used to classify the foods consumed by the adolescents according to the level of processing as: 1) *Nova group 1* - *minimally processed foods* which include natural foods that have undergone minimal processing such as milling, grinding, drying, crushing, roasting, without addition of salt, sugar or oil, 2) *Nova group 2 - foods of culinary use* which are extracted directly from group 1 foods or nature and mainly used in the cooking, preparation or seasoning of group 1 foods, e.g., oil, sugar and salt, 3) *Nova group 3 - processed foods* which include foods that have undergone processing through addition of group 2 foods (e.g., salt and sugar) mainly for preservation, improving the shelf life or sensory qualities, e.g., canned vegetables, meat, fruits, etc., and group 4) *Nova group 4 foods-UPFs* which are foods containing one or more ingredients that result from a series of industrial processes and mostly of exclusive industrial use, which are of no/rare domestic culinary use and are rarely/never used in home cooking, such as artificial flavours, sweeteners, thickeners, emulsifiers, etc. [6]. In addition to UPFs within the NOVA classification, local deep fried, savoury and sweet snacks including pastries (e.g., *doughnuts*, *mandazi*, and *samosa*) and deep fried potato snacks (e.g., French fries, *bhajia*, and crisps) purchased mainly from informal and street food vendors were identified and included in the UPFs group. This method of categorising UPFs has been used previously by Reardon et al (2021) in describing processed food typologies in sub-Saharan Africa [4]. In subsequent analysis, the UPFs group represented the less healthy food category, while the *NOVA group 1*, *2* and *3* were combined into one non-UPFs food group representing the healthier food category (Supplementary Table S1). Mean daily energy and nutrient intake from each of the two food groups; UPFs and non-UPFs, were computed by summing up the energy and nutrient intake from all the food items in each group. The caloric contribution of UPFs in daily energy intake was computed as the percentage of total energy intake (% TEI) from UPFs sources. Both mean daily energy intake (*kcal*) and % TEI from UPFs and non-UPFs were categorised into quartiles with quartile one (Q1) representing the lowest and quartile four (Q4) representing the highest intake, for subsequent analysis.

#### Nutrient Intake

The Kenya food composition tables [40] were used to establish the energy and nutrient content of the foods consumed. The nutrients assessed included total fat, saturated fat, protein, fibre, zinc, calcium and iron. Total fat and saturated fat represented nutrients associated with overweight/obesity and NCD risks while iron, calcium and zinc represented positive nutrients of concern for adolescents in Kenya [20] and the most common micronutrient deficiencies (iron and zinc) among school going children and adolescents in Kenya [25]. In cases where information on some foods or nutrients was not found in the Kenya food composition tables, other food composition tables such as Tanzania [41], Western African [42], South African [43] were consulted. Energy and nutrient information in the food composition tables is provided per 100g of each item, therefore, conversion was made to reflect the content in the actual amount consumed. The nutrient content in the diet was adjusted for energy using the energy density method; macronutrients (protein, carbohydrate, fat, saturated fat) were expressed as percentage of energy intake (% TEI) while fibre, zinc, iron and calcium were expressed per 1,000 kcal (g/mg per 1,000 kcal) [44]. Participants with energy intakes >4,000 and <500 kcal per day, indicating implausible energy intake and potential misreporting, were excluded from the analysis [45].

#### Diet Quality

The global dietary quality score (GDQS) was computed according to the standardised method by the *Intake Center for Dietary Assessment* (2022), which is validated for adolescents and women of reproductive age [46]. Food items consumed were classified into 24 food groups according to their positive or negative contribution to overall diet quality and health outcomes [46]. The 24 food groups comprised of 15 “healthy” food groups that contribute positively to overall diet quality (*citrus fruit, deep orange fruits, other fruits, dark green leafy vegetables, cruciferous vegetables, deep-orange vegetables, other vegetables, legumes, deep orange tubers, nuts/seeds, whole grains, fish and shell fish, poultry and game meat, low fat dairy, eggs*); seven “unhealthy” food groups that negatively contribute to overall diet quality (*white roots and tubers, processed meat, refined grains and baked goods, sugar-sweetened beverages, juice, sweets and ice-cream, purchased deep fried food*s); and two “*unhealthy in excessive amounts*” food groups whose optimal intake increases diet quality but excess intake decreases diet quality (*red meat, high fat dairy*). The *liquid oil* group was excluded due to difficulties in estimating the amounts and type of oil consumed by the adolescents. Mixed dishes were decomposed into the major individual ingredients while purchased deep fried snacks were double coded both in the original food group and the *purchased deep fried foods* category as described by the *Intake Centre for Dietary Assessment* (2022). Each of the food groups were assigned a score ranging from 0.25 to 4, based on the amount consumed and their contribution to diet quality [46]. The total GDQS was then calculated by summing up the scores from all the food groups consumed, with a higher GDQS indicating higher diet quality and the opposite for lower GDQS.

### Statistical Analysis

Data analysis was conducted using Stata version 17 (StataCorp, College Station, Texas United States). Descriptive statistics including mean (±sd) and percentages were used to summarise the adolescents’ socio-demographic characteristics, total energy intake, mean energy from UPFs and non-UPFs, and caloric contribution (% TEI) of UPFs to daily energy intake. Multinomial logistic regression was used to assess the factors associated with quartiles of mean energy intake from UPFs and non-UPFs (kcal/day), with quartile one (lowest intake) as the reference category for quartiles two to four (Q2, Q3, Q4) and adjusting for factors that potentially influence dietary behaviour from literature, including individual (sex, age, cultural background, leisure screen time), household (wealth index) and neighbourhood (slum of residence, duration of slum residency) characteristics.

Linear regression was used to assess the association between quartiles of %TEI from UPFs and diet quality (GDQS score), adjusting for age, sex, wealth index, slum of residence, duration of slum residency, leisure screen time, and ethnicity which showed a significant association with UPFs or non-UPFs daily energy intake. Nutrient intake data were highly skewed; therefore, Kruskal-Wallis tests were used to test the differences in median nutrient intake across the quartiles of % TEI from UPFs.

## Results

### Adolescent Characteristics by UPFs and Non-UPFs Energy Intake

A total 621 out of 660 sampled adolescents were available for the two rounds of 24-h recall interviews. Thirty-nine were unavailable for at least one round of dietary data collection due to relocation out of the study area or to boarding school. Of the n = 621, n = 14 were excluded due to implausible energy intake, whereby n = 12 reported a very low energy intake (<500 kcal per day) while n = 2 reported a very high energy intake (>4,000 kcal/day), resulting in an overall sample of n = 607 participants included in the analysis.

#### Social Demographic Characteristics

The mean (SD) age was 14 (13.7) with a slightly higher proportion of younger adolescents (63.9%) and girls (60.1%). The mean duration of stay in the slum was 12 (4.2) years, majority (72.3%) of the adolescents had lived in the slum for more than 10 years and those residing in Mathare slum (41.0%). Slightly more than half had more than 2 h of leisure screen time per day (55.2%) (Table 1).

**TABLE 1:** Adolescent characteristics and comparison of ultra-processed foods and non-ultra-processed foods energy intake by adolescent characteristics; Ultra-processed food consumption is associated with poor diet quality and nutrient intake among adolescents in urban slums, Kenya, 2021.

Individual level characteristics		Adolescents [N (%)]	Mean (SD) UPFs* energy intake (kcal/day)	Mean (SD) non-UPFs* energy intake (kcal/Day)
Sex	Male	243 (39.9)	424.0 (347.3)	1,208.8 (444.1)
	Female	364 (60.1)	429.4 (362.2)	1,150.0 (444.1)
Age (years)	10 to 14	388 (63.9)	420.7 (352.0)	1,206.2 (445.4)
	15 to 19	219 (36.1)	438.9 (363.7)	1,115.2 (438.3)
Cultural background	Kikuyu	185 (30.5)	425.3 (347.7)	1,151.8 (435.5)
	Luo	124 (20.4)	471.3 (353.3)	1,150.2 (415.4)
	Luhya	97 (16.0)	384.8 (371.5)	1,154.6 (475.3)
	Kamba	118 (19.4)	419.6 (362.0)	1,204.4 (435.9)
	Other	83 (13.7)	425.8 (353.2)	1,234.3 (483.4)
Leisure screen time	≤2 h	271 (44.8)	394.4 (347.1)	1,163.2 (453.6)
	>2 h	336 (55.2)	453.9 (361.5)	1,181.8 (437.8)
Energy intake (kcal)		607 (100)	428.1 (357)	1,175.6 (445)
Household and community level characteristics				
Wealth index quintiles	1	119 (19.7)	396.8 (310.7)	1,069.3 (435.6)
	2	123 (20.3)	398.9 (333.8)	1,134.5 (398.8)
	3	124 (20.5)	473.2 (389.1)	1,194.2 (462.5)
	4	115 (19.0)	409.7 (369.3)	1,205.7 (381.6)
	5	124 (20.5)	459.2 (370.0)	1,261.7 (513.6)
Residence	Korogocho	179 (29.5)	366.9 (283.2)	1,032.4 (359.1)
	Viwandani	179 (29.5)	397.1 (316.0)	1,297.1 (506.0)
	Mathare	249 (41.0)	492.1 (415.8)	1,185.4 (425.5)
Duration in the slum	≤10 years	168 (27.7)	483.5 (385.2)	1,186.0 (427.7)
	>10 years	439 (72.3)	405.8 (342.3)	1,168.7 (451.4)
BMI for age	Thin	38 (6.3)	465.2 (339.4)	1,090.8 (449.7)
	Overweight/obese	81 (13.3)	464.9 (367.7)	1,159.0 (484.0)
Height for age	Stunted	81 (13.3)	422.9 (389.9)	1,259.8 (435.0)

*UPFs, Ultra-processed foods.

#### Nutrition Status

A higher proportion of adolescents were overweight/obese (13.3%) compared to those who were thin (6.3%), while 13.3% were stunted (Table 1).

#### Energy Intake From UPFs and Non-UPFs

The mean (SD) daily energy intake was 1,604 (550) kcal. Mean energy from UPFs was 428 kcal contributing 25.2% of total daily energy intake. Of the UPFs energy intake, 9.1% (146 kcal) was from conventional UPFs (NOVA classification), while 17.6% (282 kcal) was from local UPFs (Table 1).

### Factors Associated With UPFs and Non-UPFs Energy Intake

#### UPFs Intake

Individual level (cultural background, screen-time) and neighbourhood characteristics (slum of residence, duration of slum residency) were associated with UPFs energy intake. Adolescents who reported a higher leisure screen time (≥2 h/day) [OR = 1.9 (95% CI 1.2–3.1)] and those living in Mathare (largest slum of the three) [OR = 2.4 (95% CI 1.3–4.3)] were more likely to have a higher UPFs energy intake (Q4) compared to those with less screen time (<2 h/day) and living in Korogocho slum, respectively. Conversely, adolescents from the Luhya cultural background [OR = 0.5 (95% CI 0.2–0.9)] and those with a longer slum residence duration (>10 years) [OR = 0.5 (95% CI 0.3–0.8)] were less likely to have higher (Q4) UPF energy intake compared to those from the Kikuyu cultural background and those with a shorter duration of stay in the slum (≤10 years), respectively (Table 2).

**TABLE 2:** Association between socio-demographic characteristics and ultra-processed foods and non-ultra-processed foods energy intake (kcal/day); Ultra-processed food consumption is associated with poor diet quality and nutrient intake among adolescents in urban slums, Kenya, 2021.

	UPFs energy intake (kcal/day)						Non-UPFs energy intake (kcal/day)					
Ref (Q1)	Q2		Q3		Q4		Q2		Q3		Q4	
	OR	95% CI	OR	95% CI	OR	95% CI	OR	95% CI	OR	95% CI	OR	95% CI
Individual characteristics												
Sex Male (ref)	1		1		1		1		1		1	
Female	1.1	(0.7–1.8)	0.9	(0.6–1.5)	1.1	(0.7–1.8)	0.7	(0.4–1.1)	0.5	(0.3–0.8)	0.7	(0.5–1.2)
Age 10–14 years	1		1		1		1		1		1	
15–19 years	0.9	(0.6–1.5)	1.2	(0.7–2.0)	1.3	(0.8–2.1)	0.8	(0.5–1.2)	1.0	(0.6–1.7)	0.5	(0.3–0.8)*
Cultural background Kikuyu (ref)	1		1		1		1		1		1	
Luo	1.1	(0.6–2.3)	1.9	(0.9–3.8)	1.3	(0.7–2.6)	0.6	(0.3–1.2)	0.7	(0.4–1.4)	1.9	(1.0–3.9)
Luhya	0.5	(0.2–1.0)	0.8	(0.4–1.6)	0.5	(0.2–0.9)*	0.8	(0.4–1.7)	0.8	(0.4–1.7)	1.3	(0.6–2.7)
Kamba	1.1	(0.6–2.1)	0.8	(0.4–1.7)	0.6	(0.3–1.1)	0.8	(0.4–1.6)	1.2	(0.6–2.3)	1.0	(0.5–2.1)
Other (Specify)	0.8	(0.4–1.8)	1.0	(0.5–2.1)	0.7	(0.3–1.4)	1.0	(0.4–2.0)	0.9	(0.4–2.1)	1.6	(0.7–3.4)
Screen time <2 h	1		1		1		1		1		1	
≥2 h	1.0	(0.6–1.6)	1.2	(0.7–1.9)	1.9	(1.2–3.1)*	1.2	(0.7–1.9)	1.6	(1–2.50)	1.2	(0.7–1.9)
Household and community characteristics												
Wealth index Poor (ref)	1		1		1		1		1		1	
Poorer	0.9	(0.4–2.0)	1.0	(0.5–2.0)	0.9	(0.4–1.9)	1.1	(0.6–2.3)	1.2	(0.6–2.5)	1.4	(0.6–3.0)
Middle	2.0	(0.9–4.3)	1.4	(0.6–2.9)	1.6	(0.8–3.5)	0.9	(0.4–1.8)	1.6	(0.8–3.2)	1.6	(0.7–3.4)
Richer	1.2	(0.6–2.6)	0.7	(0.3–1.5)	1.6	(0.4–1.8)	1.5	(0.7–3.3)	2.0	(0.9–4.3)	2.6	(1.2–5.8)*
Richest	1.2	(0.6–2.6)	1.0	(0.5–2.1)	1.6	(0.7–3.0)	1.6	(0.8–3.3)	1.0	(0.5–2.2)	2.6	(1.3–6.0)*
Slum of residence Korogocho (ref)	1		1		1		1		1		1	
Viwandani	1.0	(0.5–1.9)	1.6	(0.9–3.0)	1.5	(0.8–2.9)	0.9	(0.5–1.8)	1.8	(1–3.5)	4.4	(2.2–8.7)*
Mathare	1.0	(0.6–1.8)	1.2	(0.7–2.2)	2.4	(1.3–4.3)*	1.1	(0.6–1.8)	1.8	(1.1–3.3)	3.2	(1.7–6.0)*
Duration in the slum <10 years	1		1		1		1		1		1	
>10 years	0.7	(0.4–1.2)	0.7	(0.4–1.2)	0.5	(0.3–0.8)*	0.7	(0.4–1.2)	0.8	(0.5–1.4)	0.9	(0.5–1.5)

UPF, Ultra processed foods; OR, odds ratio. * P < 0.05

#### Non-UPFs Intake

Individual (age), household (wealth index) and neighbourhood (slum of residence) characteristics were associated with non-UPFs energy intake. Adolescents from households in the fourth [OR = 2.6 (95% CI 1.2–5.8)] and fifth [OR = 2.6 (95% CI 1.3–6.0)] wealth index quintiles were more likely to have a higher (Q4) non-UPFs energy intake compared to those in the first wealth index quintile. Adolescents living in Mathare [OR = 3.2 (95% CI 1.7–6.0)] and Viwandani [OR = 4.4 (95% CI 2.2–8.7)] were more likely to have higher non-UPFs energy intake compared to those from Korogocho (lowest SES of the three). On the other hand, older adolescents (>15 years) [OR = 0.5 (0.3–0.8)] were less likely to have a higher (Q4) non-UPF energy intake compared to younger adolescents (10–14 years) (Table 2).

### Association Between UPFs Intake, Diet Quality (GDQS) and Nutrient Intake

There was an inverse association between diet quality (GDQS) and the quartiles of % TEI from UPFs. Adolescent with the highest % TEI from UPFs (Q4) had about three points lower GDQS compared to those with the lowest % TEI from UPFs (Q1) [β −2.9 (95% CI −3.7 to −2.1)] (Table 3).

**TABLE 3:** Association between percentage energy intake from ultra-processed foods and Global Diet Quality Score; Ultra-processed food consumption is associated with poor diet quality and nutrient intake among adolescents in urban slums, Kenya, 2021.

		Quartiles of % TEI from UPFs			
Diet quality*	Mean	Q1	Q2	Q3	Q4
		ref	β(95% CI)	β(95% CI)	β(95% CI)
GDQS	20.5 (18.5–23.0)	1	−1.3 (−2.1–−0.5)	−1.9 (−2.7–−1.1)	−2.9 (−3.7–−2.1)

*Regression analysis of association between GDQS, score and quartiles of % TEI from UPFs adjusting for age, sex, wealth, slum, ethnicity, duration in slum, leisure screen time.

GDQS, global diet quality score; UPFs, Ultra processed foods.

Median energy, total fat and saturated fat increased while protein, fibre, calcium and zinc decreased across the quartiles of % TEI from UPFs. Adolescents with the highest % TEI from UPFs (Q4) had the highest median total energy, total fat and saturated fat intake; and lowest median proteins, fibre, iron, zinc and calcium intake (Table 4).

**TABLE 4:** Median nutrient intake across quartiles of percentage energy intake from ultra-processed foods; Ultra-processed food consumption is associated with poor diet quality and nutrient intake among adolescents in urban slums, Kenya, 2021.

Nutrient intake*	Quartiles of % TEI from UPFs					*P*-value
	Median	Q1	Q2	Q3	Q4	
Energy (kcal)	1,543.4 (1,207.7–1,912.2)	1,345.7	1,501.9	1,551.9	1,771.1	0.001
Fat (%TEI)	26.2 (22.0–30.5)	24.3	24.7	26.1	28.4	0.001
Saturated fat (% TEI)	8.8 (6.1–12.6)	7.7	8.0	9.1	10.4	0.001
Carbohydrate (% TEI)	59.9 (55.4–64.0)	60.2	60.9	60.0	58.9	0.011
Protein (% TEI)	10.8 (9.8–12.3)	11.9	11.1	10.8	9.8	0.001
Fibre (mg/1000 kcal)	19.0 (15.3–22.9)	22.6	20.2	18.6	15.5	0.001
Iron (mg/1,000 kcal)	9.4 (8.2–11.2)	9.6	9.3	9.5	9.2	0.153
Zinc (mg/1,000 kcal)	4.1 (3.6–4.8)	4.8	4.4	4.0	3.4	0.001
Calcium (mg/1,000 kcal)	352.1 (238.7–408.8)	344.7	334.0	335.7	282.2	0.001

*Kruskal-Wallis test of the difference in the median nutrient intake across the quartiles of UPFs energy.

*UPFs, Ultra-processed foods.

## Discussion

This study assessed the caloric contribution of UPFs to daily energy intake, the factors associated with UPFs and non-UPFs energy intake and the relationship between UPFs energy intake, diet quality and nutrient intake among adolescents in urban slums in Nairobi, Kenya. The findings indicate that about a quarter of adolescents’ daily energy is from unhealthy (UPFs) food sources. Individual (age, ethnicity and screen time), household (wealth index), and community/neighbourhood factors (slum of residence and duration of stay) are associated with UPF/non-UPFs energy intake. Adolescents with high UPFs energy intake are likely to have a poor overall diet quality, high intake of nutrients promoting obesity/NCDs (total fat and saturated fats) and lower intake of health promoting nutrients (protein, fibre, calcium, iron, zinc). Overweight/obesity was more prevalent than thinness. About 13% of the adolescents were overweight/obese, aligning with the national prevalence of overweight/obesity (14%) among adolescents, while the prevalence of thinness (6%) was slightly lower than the national average (13%) [24].

Most of adolescents’ daily energy intake comes from non-UPFs sources which aligns with the Kenyan healthy eating guidelines/key messages encouraging the consumption of unprocessed or minimally processed foods and limiting the consumption of processed foods and those high in fat, salt and sugar [20]. This also agrees with global literature indicating high consumption of non-processed or minimally processed foods, such as grains, fruit, and vegetables and lower consumption of UPFs in SSA compared to other parts of the world, especially in HICs [4, 47, 48]. UPFs (NOVA classification) contribution to daily energy intake (9%) by adolescents in this study aligned with findings from previous studies in Kenya (8%) [49] and Ethiopia (9%) [50], although both studies did not include locally prepared UPFs. The UPFs consumption was much lower than from middle and high income countries such as Brazil, Belgium and the UK where UPFs contribution to adolescents daily energy intake is about 30% and 60% [9, 51, 52]. However, with the on-going nutrition transition observed in LMICs including SSA, UPFs consumption is projected to soon equal that in HICs, if no mitigation plans are undertaken [4, 53]. This is of concern given adverse health outcomes, such as overweight, obesity, cardiometabolic, mental and neurological conditions that have been linked to high UPF consumption in adolescents in HICs [7, 54–56]. It is also important to note that in addition to industrially produced UPFs as described in the NOVA classification, locally prepared UPFs contribute substantially to unhealthy food consumption among adolescents in the study context. Turner et al. pointed out the existence of informal food systems in LMICs such as street vendors who provide local ready to eat, cheap street fast foods, that have minimum or no food packaging and labelling, as a key difference between food environments in HICs and LMICs [16]. Implementation of food environment policies to mitigate unhealthy food consumption in LMICs should therefore address the wide range of unhealthy food types supplied through both formal (pre-packaged UPFs) and informal food (local prepared UPFs) systems.

We found that adolescents’ individual, household and community/neighbourhood characteristics were associated with UPFs consumption. At an individual level, screen time was associated with higher UPFs consumption. Similarly, a high screen time was associated with higher UPFs consumption in other studies [57, 58], some concluding that prolonged television and computer viewing hours favoured the passive consumption of junk foods and sugar sweetened beverages [59]. In a qualitative study among adolescents in the study area watching TV and spending time on social media were highlighted as among the competing activities that hindered the preparation and consumption of healthy homemade meals, leading adolescents to opt for more convenient UPFs that were ready to eat or needed minimal preparation [60]. As such, interventions to promote healthier dietary behaviour for adolescents should incorporate strategies to limit leisure screen time in favour of health promoting activities, such as play and physical activity.

At household level, higher wealth index quintile was associated with non-UPFs consumption. Similarly, at neighbourhood level, adolescents living in the (relatively) higher SES slum (Viwandani) were more likely to consume non-UPFs compared to those in the poorest slum (Korogocho). Our finding aligns with those from other studies that have found associations between UPFs consumption and socio-economic status, with a higher caloric cost of non-UPFs foods compared to UPFs [61] and higher likelihood of UPFs consumption among individuals and neighbourhoods with low socio-economic situation [62, 63]. A study of food insecurity in urban slums Nairobi indicated consumption of cheap, ready-to-eat and street foods as a strategy to save food related costs, compared to preparation of home-made meals requiring extra expenses for preparation and cleaning such as water and fuel [64]. This was also reflected in a qualitative study with adolescents in the study area which revealed economic access as one of the drivers of UPFs consumption, with a general perception that UPFs were cheap and easily affordable in the slum neighbourhood while non-UPFs were less affordable [60]. This may explain the higher likelihood of consumption of non-UPFs by wealthier households and higher SES neighbourhoods than poorer households/neighbourhoods. Strategies to create a healthier food environment in urban contexts should therefore consider improving the affordability and accessibility of healthier non-UPFs in economically deprived neighbourhoods and households.

Our study demonstrates that high-energy intake from UPFs sources is linked to poor diet quality (lower GDQS). This concurs with studies in Ethiopia and Brazil where UPFs energy intake was inversely correlated with diet quality (GDQS) [50, 65], and a multi-country European study where UPFs consumption was associated with poor diet quality, lower fruits and vegetables consumption and high consumption of “junk” foods [11].

Furthermore, higher UPFs caloric intake was related to higher consumption of nutrients related to obesity and NCDs (total fat, saturated fat) and decreased intake of health promoting nutrients (protein, fibre, iron, calcium, zinc) by the adolescents. Similarly in Brazil, high energy contribution from UPFs was associated with lower protein, fibre, iron and zinc intake in young adolescents [10], while in Chile, UPFs consumption was associated with high fat and saturated fat intake and inversely associated with fibre intake [66]. UPFs consumption therefore is detrimental to achieving healthy diets and optimal diet quality and nutrient intake by adolescents, which potentially increases their susceptibility to overweight/obesity, micronutrient deficiencies and diet related NCDs in the long-term [46, 67]. As such, efforts to address the double burden of malnutrition and chronic micronutrient deficiencies among adolescents in SSA should include the reduction in UPFs consumption as one of the strategies.

### Strengths and Limitations

This is the first study to provide evidence on the extent of consumption of UPFs, factors associated with UPFs consumption and the association between UPFs, diet quality and nutrient intake among adolescents in urban slum contexts in Kenya. The study combined both industrially produced UPF as described in the NOVA system and also local UPFs produced and supplied through informal and street food vendors, ensuring a comprehensive inclusion of the wide range of unhealthy foods consumed by adolescents in Kenyan urban slum contexts. The use of a 24-h recall limits the assessment of usual UPFs intake by the adolescents. Therefore, longitudinal studies are recommended to track the consumption of UPFs by adolescents and its association with health outcomes.

### Conclusion

Unhealthy foods, including UPFs, substantially contribute to adolescents’ daily energy intake, and are related to poor diet quality, lower intake of health promoting and higher intake of obesity and NCD related nutrients. This calls for interventions to address the consumption of unhealthy foods among adolescents in urban slums in Kenya and SSA. Such interventions should incorporate the wide range of unhealthy food types supplied through both formal and informal food environment in Kenyan urban slums and similar contexts.
