# Supplementary material for: Ultra-Processed Food Consumption Is Associated With Poor Diet Quality and Nutrient Intake Among Adolescents in Urban Slums, Kenya
Source: Int J Public Health. 2025 Mar 5;69:1607891. doi: 10.3389/ijph.2024.1607891 (PMC11919627; doi:10.3389/ijph.2024.1607891)
Supplement: Supplementary file 1 [file Table1.docx]

**SUPPLEMENTARY FILES**

**Ultra-processed food consumption is associated with poor diet quality and nutrient intake among adolescents in urban slums, Kenya, 2021**

Supplementary Table 1: Food classification according to NOVA classification and local UPFs

| **UNPROCESSED /MINIMALLY PROCESSED** | |  |  |  |  |
| --- | --- | --- | --- | --- | --- |
|  |  |  |  |  |  |
| **FOOD GROUP** | **FOOD ITEM** | **FOOD GROUP** | **FOOD ITEM** |  |  |
| Whole grains and cereals *wholemeal (brown) chapati* | |  |  |  |  |
|  | *white injera* | Egg | *egg* |  |  |
|  | *brown injera* |  | *omelette* |  |  |
|  | *wholemeal ugali* |  |  |  |  |
|  | *boiled maize* | Red and organ meat | *beef* |  |  |
|  | *roasted maize* |  | *goat* |  |  |
|  | *local brown rice* |  | *liver* |  |  |
|  |  |  | *mutura* |  |  |
| Refined grains and cereals | *white chapati* |  | *bone soup* |  |  |
|  | *white ugali* |  | *meat stew* |  |  |
|  | *refined porridge* |  | *offals (matumbo)* |  |  |
|  | *pancake* |  |  |  |  |
|  | *white rice* | Chicken | *chicken giblets* |  |  |
|  | *pasta* |  | *chicken* |  |  |
|  | *macaroni* |  |  |  |  |
|  |  | Fish | *omena* |  |  |
| Homemade mixed dishes | *githeri* |  | *fish* |  |  |
|  | *muthokoi* |  | *dried fish* |  |  |
|  | *mukimo* |  |  |  |  |
|  | *pilau* | Fresh fruit juice | *freshly prepared fruit juices* |  |  |
|  | *biryani* |  |  |  |  |
|  | *rice and potatos* | Hot beverages (Homemade) | *unsweetened tea with milk* |  |  |
|  | *rice and beans* |  | *sweetened tea with milk* |  |  |
|  | *vegetable sandwiches* |  | *sweetened tea without milk* |  |  |
|  | *tomato soup* |  | *sweetened coffee with milk* |  |  |
|  | *vegetable stew* |  | *sweetened coffee without milk* |  |  |
|  | *toasti mayai* |  | *unsweetened cocoa/milo with milk* |  |  |
|  |  |  | *unsweetened cocoa/milo without milk* |  |  |
| Milk and Dairy | *milk* |  | *sweetened cocoa/milo with milk* |  |  |
|  | *fermented milk (maziwa mala mursik) cream* |  | *sweetened cocoa/milo without milk* |  |  |
|  |  |  |  |  |  |
| Fresh fruits | *ripe mango* | **ULTRAPROCESSED FOODS (UPFs)** |  |  |  |
|  | *ripe pawpaw* |  |  |  |  |
|  | *passion fruit* | Industrial bread |  |  |  |
|  | *tree tomato* |  | *whole (brown) bread* |  |  |
|  | *dairy fruit* |  | *anyona* |  |  |
|  | *watermelon* |  | *buns* |  |  |
|  | *pineapple* |  | *white bread* |  |  |
|  | *banana* |  | *scone* |  |  |
|  | *apple* |  |  |  |  |
|  | *avocado* | Cake and biscuits | *cake* |  |  |
|  | *lemon* |  | *cream pie* |  |  |
|  |  |  | *biscuits cookies* |  |  |
| Starchy roots/tubers | cassava |  |  |  |  |
|  | *potatos* | potato snacks | *crisps* |  |  |
|  | *sweet potatos (white)* |  | *chips (snack made from flour dough fried)* |  |  |
|  | *yam* |  |  |  |  |
|  | *arrowroots* | Instant noodles | *instant noodles* |  |  |
|  | *bananas* |  |  |  |  |
|  | *sweet potatos (yellow or orange inside)* | Flavoured yoghurt | *flavoured yoghurt* |  |  |
|  | *squash* |  |  |  |  |
|  | *pumpkin* | Processed meats | *hot dog* |  |  |
|  | *stwed potatos* |  | *smokies* |  |  |
|  |  |  |  |  |  |
| Fresh vegetables | *carrots* | Sweets and confectionery | *salted popcorn* |  |  |
|  | *tomatoes* |  | *corn puffs* |  |  |
|  | *red or yellow pepper (capsicum)* |  | *sweetened popcorn* |  |  |
|  | *kales* |  | *chocolate* |  |  |
|  | *sukuma wiki* |  | *sweets and toffee* |  |  |
|  | *managu* |  | *mabuyu* |  |  |
|  | *terere* |  | *ice cream* |  |  |
|  | *saga* |  |  |  |  |
|  | *mrenda* | Sugar sweetened beverages | *sodas* |  |  |
|  | *pumpkin leaves* |  | *fruit based drinks (lucozade etc.)* |  |  |
|  | *osuga/sucha* |  | *cordials* |  |  |
|  | *kunde* |  | *squashes* |  |  |
|  | *other traditional vegetables* |  | *commercial fruit juices (sweetened)* |  |  |
|  | *spinach* |  |  |  |  |
|  | *green leaves* | Magarine | *margarine* |  |  |
|  | *spinach* |  |  |  |  |
|  | *lettuce* | Sauces and ketchup | *tomato and chilli sauce (ketchup)* |  |  |
|  | *cabbage* |  |  |  |  |
|  | *eggplant* |  |  |  |  |
|  | *mixed vegetable salad* | **LOCAL UPFS** |  |  |  |
|  | *green beans* |  |  |  |  |
|  | *french beans* | Local deep fried pastry | *doughnut* |  |  |
|  | *green peas (minji)* |  | *mandazi* |  |  |
| Legume/pulses | *wholemeal porridge (Millet)* |  | *gumu* |  |  |
|  | *beans* |  | *samosa* |  |  |
|  | *lentils* | Deep fried potato snacks | *chips/french fries* |  |  |
|  | *peas* |  | *bhajia* |  |  |
|  | *soya beans* |  |  |  |  |
|  | *beans* |  |  |  |  |
|  | *green grams (ndengu)* |  |  |  |  |
|  | *mbaazi* |  |  |  |  |
|  | *njahi* |  |  |  |  |
|  | *groundnuts* |  |  |  |  |
